# Channel-Supermodular Entropies: Order Theory and an Application to Query Anonymization †

**DOI:** 10.3390/e24010039

**Published:** 2021-12-25

**Authors:** Arthur Américo, MHR Khouzani, Pasquale Malacaria

**Affiliations:** School of Electronic Engineering and Computer Science, Queen Mary University of London, London E1 4NS, UK; a.passosderezende@qmul.ac.uk (A.A.); arman.khouzani@qmul.ac.uk (M.K.)

**Keywords:** information theory, quantitative information flow, channel ordering, more-capable, less-noisy, broadcast channels, anonymity

## Abstract

This work introduces channel-supermodular entropies, a subset of quasi-concave entropies. Channel-supermodularity is a property shared by some of the most commonly used entropies in the literature, including Arimoto–Rényi conditional entropies (which include Shannon and min-entropy as special cases), k-tries entropies, and guessing entropy. Based on channel-supermodularity, new preorders for channels that strictly include degradedness and inclusion (or Shannon ordering) are defined, and these preorders are shown to provide a sufficient condition for the more-capable and capacity ordering, not only for Shannon entropy but also regarding analogous concepts for other entropy measures. The theory developed is then applied in the context of query anonymization. We introduce a greedy algorithm based on channel-supermodularity for query anonymization and prove its optimality, in terms of information leakage, for all symmetric channel-supermodular entropies.

## 1. Introduction

The idea of preorders over channels goes back a long way in the history of information theory. For instance, in [1], Shannon introduced the “*inclusion*” preorder to compare the capacities of discrete memoryless channels. Several authors, such as El Gamal [2], Korner and Marton [3], and many more, made further significant contributions to the study of channel preorders.

Such preorders are of practical importance in information theory. For example, the “*more capable*” preorder [3] is used in calculating the capacity region of broadcast channels [2], or in deciding whether a system is more secure than another [4,5]. As discussed in the book by Cohen, Kempermann, and Zbaganu [6], the applications of preorders over stochastic matrices goes beyond the field of information theory, for instance, to statistics, economics, and population sciences.

In this work, which is an extension of the results in our previous work [7], we introduce a new preorder over channels. To illustrate the key idea, consider the following channel:

$$\left(\begin{array}{ccc} 0.3 & 0.5 & 0.2\\ 0.6 & 0.4 & 0\\ 0.2 & 0.3 & 0.5 \end{array}\right).$$

Now build a new channel from it as follows: take the first two columns, and for each row, rearrange their pairwise entries such that the larger element is moved to the first column, and the smaller element is in the second column. This yields:

$$\left(\begin{array}{ccc} 0.5 & 0.3 & 0.2\\ 0.6 & 0.4 & 0\\ 0.3 & 0.2 & 0.5 \end{array}\right).$$

We will refer to this pairwise operation on columns as a *Join-Meet* operation. We prove that, for most commonly used entropy measures, a *Join-Meet* operation always increases the posterior entropy. More precisely, the posterior entropy of the derived channel is never less than the posterior entropy of the original channel, for any probability distribution on the input. That is, the original channel is *more capable* [3] than the derived channel. We name the entropies respecting this property *channel-supermodular*, and prove they entail Arimoto–Rényi entropies (including Shannon and min-entropy), and the guessing entropy, as well as some other entropies that are motivated from security and privacy contexts.

We define the *supermodular* preorder ($\geq_{s}$) based on the Join-Meet operator. In particular, given channels $K_{1}$ and $K_{2}$, we say $K_{1}\geq_{s}K_{2}$ iff $K_{2}$ can be obtained from $K_{1}$ via a finite sequence of Join-Meet operations. We establish that the supermodular preorder is neither included nor does it include the *degradedness* [8] or *inclusion* (Shannon) [1] preorders. Motivated by this, we define two other channel preorders ($\geq_{\mathrm{ds}}$ and $\geq_{\mathrm{shs}}$) that strictly include the aforementioned ones, respectively. The relation $K_{1}\geq_{\mathrm{ds}}K_{2}$ implies that $K_{1}$ is “more capable” than $K_{2}$. Moreover, whenever $K_{1}\geq_{\mathrm{shs}}K_{2}$, then the capacity of $K_{1}$ is higher than that of $K_{2}$. Several such new channel ordering results are proven in this paper based on channel-supermodularity.

Next, we will consider the applications of channel-supermodularity in the context of security and privacy. The starting point will be the channel design problem, which is the problem of designing a channel that leaks the least amount of confidential information while respecting a set of operational constraints. This kind of problem arises in many security systems, such as authentication systems [9], operating systems functions [10], scheduling protocols [11], bucketing schemes in cryptography [12], anonymity (Section 6.2), and so on. In the context of these applications, the problem is particularly interesting for deterministic systems and deterministic solutions. Solutions which are unique across many measures of leakage are also of interest because they are robust against how the knowledge or abilities of the attackers are modeled. In this work, we present a robust anonymity mechanism. The algorithm is based on a result from [13], which uses the properties of channel-supermodularity presented in this paper to derive a greedy channel design algorithm that is provably optimal and unique for all channel-supermodular measures of leakage. We apply our robust anonymity mechanism to query anonymization: we consider the problem in which the real query itself is the secret, and we also consider the scenario where a related attribute is the secret. We provide optimal solutions for these two problems based on channel-supermodularity.

### 1.1. Related Literature

In the “information theory” literature, the degradedness order was introduced by Cover [8] in the study of broadcasting channels. Cover conjectured a solution for the capacity region of broadcast channels that satisfy the degradedness ordering, which was proved by Bergmans [14,15] and Gallager [16]. The problem of determining the capacity region of broadcast channels also motivated Korner and Marton to introduce the $H_{1}$-less noisy and $H_{1}$-more capable orderings [3]. In the same paper, Korner and Marton established the capacity region for broadcast channels that respect the $H_{1}$-less noisy ordering. A similar result for broadcast channels respecting the $H_{1}$-more capable ordering was later established by El Gamal [2].

Those orderings also play an important role in the field of *quantitative information flow* (QIF), which is concerned with quantifying information leakage in computational systems (we refer to [17] for a review of QIF). Malacaria [18] makes use of degradedness ordering to reason about the security of deterministic programs, proving it is equivalent to the $H_{1}$, $H_{\infty }$, and $H_{G}$-more capable orderings for deterministic channels. This ordering also appears in the work of Alvim et al. [19], in which it is shown to imply the more capable ordering for the *g-entropy* family, a generalizing framework for information measures used in QIF. In the same paper, they conjectured that if two channels satisfy the more capable ordering for all members of the *g-entropy* family, they satisfy the degradedness order. This conjecture, which was proven by McIver et al. [20], turns out to be equivalent to Blackwell’s theorem in the finite setting [21]. The less noisy ordering also appears recently in QIF literature, especially the $H_{\infty }$-less noisy ordering, in the study of *Dalenius leakage* by Bordenabe and Smith [22]. This last work is also closely related to the classical implications of the work by Buscemi [23], which is mainly focused on the $H_{\infty }$-less noisy ordering in quantum information theory.

Shannon ordering (or inclusion), which generalizes degradedness ordering, was first introduced by Shannon when studying channels that could be perfectly simulated by other channels [1]. In the same paper, Shannon established that inclusion implies $H_{1}$-capacity ordering. This ordering has been the object of study of several recent works. Inspired by Le Cam’s concept of deficiency [24], which is itself related to the degradedness order and Blackwell’s theorem, Raginsky [25] defines *Shannon deficiency*, which may be seen as a measure of how far two channels are from satisfying the inclusion order. Techniques to verify Shannon ordering, both algebraic and computational, were studied by Zhang and Tepedelenlioǧlu [26], and Nasser [27] gave two different characterizations of the Shannon ordering.

The abstract problem of designing a system that leaks the least amount of information under some generalized form of operational constraints has been the objective of recent exploration in the literature [28,29,30]. The problem of optimal system design in security settings has been studied within specific contexts, including secure multi-party computation systems [31] and countermeasures against timing attacks [12,32]. The general channel design problem is of particular significance in QIF, as it represents a paradigm shift from the earlier foundational research in the area, which focuses mostly on measuring information leakage for existing channels or systems [4,5,19,33,34].

Query obfuscation and anonymity have been investigated by several authors and even implemented in commercial products. Related to our work are algorithms presented in [35,36,37]. Compared to those works, our approach follows an order-theoretical methodology and is based on a more general notion of entropy.

All the results in this paper rely on the concept of core-concave entropies, a generalizing framework that has been recently developed and can be shown to generalize the most commonly used conditional entropy measures in the literature [28]. Core-concavity is a generalization of concavity, which has also been considered as a defining or desirable property for generalized information measures in QIF [38] and in information theory [39,40,41,42].

### 1.2. Notational Conventions

Throughout the paper, X,Y,Z,… represent discrete random variables with (nonempty, finite) alphabets X,Y,Z,…. We assume that the elements of each alphabet are ordered, denoting by $x_{1},\ldots ,x_{\left|X\right|}$ the elements of X, by $y_{1},\ldots ,y_{\left|Y\right|}$ the elements of Y, and so on. Given $x_{i}\in X$, we write $p(x_{i})$ or $p_{i}$ to mean $Pr\{X=x_{i}\}$, and use *p* to refer to the (categorical) distribution (as a vector). We may specify the r.v. with a subscript, for example, writing $p_{X}\left(x\right)$, if it is not clear from the context.

We denote by $\Delta_{n}\subset R^{n}$ the (n−1)-dimensional probability simplex. Given a probability distribution *p* over $\left\{x_{1},\ldots ,x_{n}\right\}$, we overload the notation and use *p* to refer to its probability vector $\left(p_{1},\ldots ,p_{n}\right)\in \Delta_{n}$. We write $\left(p_{\left[1\right]},p_{\left[2\right]},\ldots ,p_{\left[n\right]}\right)$ for the nonincreasing rearrangement of $p=(p_{1},\ldots ,p_{n})$—that is, $p_{\left[1\right]}$ denotes largest element of *p*, $p_{\left[2\right]}$ the second largest, and so forth. Given a vector $r=\left(r_{1},\ldots ,r_{n}\right)\in R^{n}$, we denote by ${∥r∥}_{\alpha }$ its α-norm ${\left(\sum r_{i}^{\alpha }\right)}^{1\;/\;\alpha }$ (note that this is a slight abuse of nomenclature, since it is not a norm when α<1).

Given a function *F* over $\Delta_{n}$ and a random variable *X* with distribution $p=(p_{1},\ldots ,p_{n})$, we use F(X), $F(p_{1},\ldots ,p_{n})$ and F(p) interchangeably.

A channel *K* is a row stochastic matrix with rows indexed by X and columns indexed by Y. The value K(x,y) is equal to p(y|x)=Pr{Y=y|X=x}, that is, the conditional probability that *y* is produced by the channel *K* when *x* is the input value. The notation K:X→Y means that the channel *K* has X and Y as input and output alphabets, respectively.

$$p\left(x,y\right)=p_{X}\left(x\right)K\left(y|x\right).$$

Channels are represented in a table format, or simply in the matrix notation, for  example:

$$\begin{array}{ccc} K & y_{1} & y_{2}\\ x_{1} & 0.5 & 0.5\\ x_{2} & 0.6 & 0.4\\ x_{3} & 0.2 & 0.8 \end{array}\;,\left(\begin{array}{cc} 0.5 & 0.5\\ 0.6 & 0.4\\ 0.2 & 0.8 \end{array}\right),$$

with the understanding that the *i*th row corresponds to $x_{i}$, and the *j*th column to $y_{j}$.

## 2. Preliminaries

### 2.1. Core-Concave Entropies

The main result this work provides is the monotonicity of conditional entropy with regard to the Join-Meet operator, which will be introduced in Section 3. This holds not only for Shannon entropy, but also for a number of different entropy measures, including the Arimoto–Rényi entropies (which include Shannon and min-entropy as limit cases) [43], and the guessing entropy [44].

Most of the results in this paper concern the aforementioned entropies, which are instances of what we call *channel-supermodular* entropies. To define a channel supermodular entropy, however, we first need a generalizing framework. To this end, we introduce the core-concave entropies based on the framework introduced in [28]. Besides the ones aforementioned, they also include the Tsallis [45] and Sharma–Mittal [46] entropies.

**Definition** **1.** *A “core-concave” entropy H is a pair (η,F) such that*

- *$F:\Delta_{n}\to R$ is a concave and continuous function,*
- *η is a strictly increasing continuous real-valued function, defined on the image of the function F.*

*Given a core-concave H=(η,F), we define $H\left(X\right)=\eta (F\left(p_{X}\right))$. The set of core-concave entropies will be denoted by H.*

Besides the value H(X), which we refer to as the *unconditional form*, we also define a conditional form for the core-concave entropies, with relation to two random variables X,Y.

**Definition** **2.** *The “conditional form” of a core-concave entropy H=(η,F) is defined as*

$$\begin{array}{c} H\left(X|Y\right)=\eta \left(\sum_{y\in \mathrm{supp}(Y)}p\left(y\right)F\left(X|y\right)\right) \end{array}$$

*where supp(Y) is the support of Y.*

As claimed before, core-concave entropies encompass the most common entropies in the literature. Some of these are summarized in Table 1, together with their conditional form. Notice that *H* is used to denote an arbitrary core-concave entropy, while $H_{1}$ refers to Shannon entropy.

Based on Definitions 1 and 2, we define a notion of mutual information and channel capacity for each H∈H.

**Definition** **3.** *Let H∈H. The H-mutual information is defined as*

$$I_{H}\left(X;Y\right)=H\left(X\right)-H\left(X|Y\right).$$

*When X and Y are respectively the input and output of a channel K, the H-channel capacity of K is defined as*

$$C_{H}\left(K\right)=\max_{p_{X}}I_{H}\left(X;Y\right).$$

Notice that, in general, *H*-mutual information is not symmetric.

Core-concave entropies satisfy the data-processing inequality.

**Theorem** **1** ([28] Proposition 2(b)). *Let X,Y,Z be random variables such that X→Y→Z (i.e., X and Z are conditionally independent given Y). Then, for all H∈H,*

H(X|Y)≤H(X|Z).

### 2.2. Preorders over Channels

Let $K_{1}:X\to Y$, $K_{2}:X\to Z$ be channels which share an input *X* and produce outputs Y,Z.

A channel $K_{2}$ is *degraded from* $K_{1}$ [8], written as $K_{1}\geq_{d}K_{2}$, if there exists a channel R:Y→Z such that $K_{2}=K_{1}R$.

In [1], Shannon introduced a preorder which includes the one above. A channel $K_{1}$ *includes* $K_{2}$, written as $K_{1}\geq_{\mathrm{sh}}K_{2}$, if there exists a family of tuples ${\left\{\left(g_{i},T_{i},R_{i}\right)\right\}}_{i}$ of channels $T_{i},R_{i}$ and non-negative real numbers $g_{i}$ such that:

(1) $$\begin{array}{c} K_{2}=\sum_{i}g_{i}T_{i}K_{1}R_{i}\mathrm{and}\sum_{i}g_{i}=1. \end{array}$$

As noted by Shannon, any channel can be expressed as a convex combination of deterministic channels. Thus, whenever $K_{1}\geq_{\mathrm{sh}}K_{2}$, it is possible to choose ${\left\{\left(g_{i},T_{i},R_{i}\right)\right\}}_{i}$ such that $T_{i},R_{i}$ are deterministic channels and (1) holds.

The two preorders defined above are not dependent on a particular entropy but on the structure of the channel. The next preorders depend on the choice of a core-concave entropy *H* and generalize preorders introduced in [3].

A channel $K_{1}$ is *H-less noisy* than $K_{2}$, denoted as $K_{1}\geq_{\ln}^{H}K_{2}$, if for all random variables *U* with finite support such that U→X→(Y,Z),

$$\begin{array}{c} I_{H}\left(U;Y\right)\geq I_{H}\left(U;Z\right). \end{array}$$

$K_{1}$ is *H-more capable* than $K_{2}$, written as $K_{1}\geq_{\mathrm{mc}}^{H}K_{2}$, if for all distributions of the input, *X*,

$$\begin{array}{c} I_{H}\left(X;Y\right)\geq I_{H}\left(X;Z\right). \end{array}$$

Finally, $K_{1}\geq_{c}^{H}K_{2}$ stands for

$$C_{H}\left(K_{1}\right)\geq C_{H}\left(K_{2}\right).$$

If A⊂H is a subset of core-concave entropies, $K_{1}\geq_{\ln}^{A}K_{2}$ is defined as:

$$\forall H\in A,\;K_{1}\geq_{\ln}^{H}K_{2}.$$

This is similar for $\geq_{\mathrm{mc}}^{A}$ and $\geq_{c}^{A}$.

### 2.3. Relationships between Preorders

We now explore some relationships between the preorders defined above.

**Proposition** **1.** *For any H∈H:*

$$\begin{array}{c} K_{1}\geq_{d}K_{2}\Rightarrow K_{1}\geq_{\ln}^{H}K_{2}\Rightarrow K_{1}\geq_{\mathrm{mc}}^{H}K_{2}\Rightarrow K_{1}\geq_{c}^{H}K_{2}. \end{array}$$

**Proof.**  The first implication follows from Theorem 1, the second implication follows by choosing U=X in the definition of $\geq_{\ln}^{H}$, and the third is straightforward.    □

While the reverse of the above implications is false, the following is true:

**Theorem** **2** ([23]). *$K_{1}\geq_{\ln}^{H_{\infty }}K_{2}\Rightarrow K_{1}\geq_{d}K_{2}$.*

The following important theorem can be traced back to Blackwell’s result on comparison of experiments [21] and relates to more recent results in [20,48].

**Theorem** **3.** *$K_{1}\geq_{d}K_{2}\Leftrightarrow K_{1}\geq_{\mathrm{mc}}^{H}K_{2}$.*

## 3. Channel-Supermodular Entropies

Note that any specific core-concave entropy *H* induces a *H*-more capable preorder over channels. However, this preorder might not be preserved for a different choice of conditional entropy. Theorem 3 characterizes a channel preorder that is “consistent” for all core-concave entropies. As strong as this result is, it still leaves the question whether there exists a preorder that is consistent for a class of entropies of interest. This is motivated by the fact that the class of core-concave entropies include far more entropies than the conventionally used ones, so it may include some eccentric ones that can be excluded for a stronger result. Moreover, the degradedness relation between channels seems very restrictive: there are many channels that cannot be compared with respect to degradedness, but have consistent ordering with respect to all typically used entropies.

With these motivations in mind, we introduce *channel-supermodular entropies*. Channel-supermodularity is a property satisfied by a significant portion of commonly used entropies, being a helpful tool in optimization problems (as shown in Section 6.1).

The characterization of channel-supermodular entropies is linked to *supermodular functions* over the real lattice. These functions and some basic properties are introduced next. For details about supermodular functions please refer to [49] and [50] (Chapter 6.D).

Consider the set $R_{\geq 0}^{n}$ of all *n*-dimensional vectors with no negative entries (i.e., the non-negative orthant of $R^{n}$). Let ⪯ represent the element-wise inequality, that is, given $r=(r_{1},\ldots ,r_{n})$ and $s=(s_{1},\ldots ,s_{n})$, r⪯s iff $r_{i}\leq s_{i}$ for all *i*. ⪯ is a partial order on $R_{\geq 0}^{n}$. In fact ⪯ defines a lattice over $R_{\geq 0}^{n}$, whose join ∨ and meet ∧ operations are defined as:

$$\begin{array}{c} r\vee s=(\max\left(r_{1},s_{1}\right),\ldots ,\max\left(r_{n},s_{n}\right)),\\ r\wedge s=(\min\left(r_{1},s_{1}\right),\ldots ,\min\left(r_{n},s_{n}\right)). \end{array}$$

Recall that:

**Definition** **4.** *A function $\phi :R_{+}^{n}\to R$ is* supermodular *(over a lattice) if, for all $r,s\in R_{+}^{n}$,*

ϕ(r∨s)+ϕ(r∧s)≥ϕ(r)+ϕ(s).

Next, we introduce some fundamental definitions for this work:

**Definition** **5.** *Let H=(η,F) be a core-concave entropy. Define the function $G_{F}:R_{\geq 0}^{n}\to R$ as*

$$G_{F}\left(r\right):={∥r∥}_{1}F\left(\frac{r}{{∥r∥}_{1}}\right)$$

*if r is not the null vector, and $G_{F}\left(0,\ldots ,0\right)=0$. (Notice that, as F is continuous over a compact set, $\lim_{r\to (0,\ldots ,0)}G_{F}\left(r\right)=0$.)*

**Definition** **6.** *An entropy H=(η,F)∈H is said to be* channel-supermodular *if $G_{F}$ is supermodular. The set of channel-supermodular entropies is noted by S⊂H.*

The motivation for defining channel-supermodularity in terms of $G_{F}$ might seem arbitrary, but it is justified for its relationship with conditional entropies, given by

(2) $$H\left(X|Y\right)=\eta \left(\sum_{y\in Y}G_{F}\left(p_{\left(X,Y\right)}\left(x_{1},y\right),\ldots ,p_{\left(X,Y\right)}\left(x_{n},y\right)\right)\right).$$

Together with (2), the supermodularity of $G_{F}$ can be a powerful tool for deriving results regarding conditional entropy and mutual information for entropies in S.

### 3.1. Examples of Channel-Supermodular and Non-Channel-Supermodular Entropies

In the next sections, we will study the implications of channel-supermodularity. The inequality in Definition 4 implies interesting behaviors regarding *H*-mutual information, as will be seen in Section 4. This property has immediate consequences for channel ordering and channel design, as will be explored in Section 5 and Section 6.

One appealing aspect of channel-supermodularity is that some of the most commonly used entropies in the literature belong to S, including Shannon and min-entropy, and more generally the Arimoto–Rényi entropies. In this section, we prove that these (and other entropies) indeed belong to S, and provide examples of entropies that do not. First, we state a useful characterization of supermodular functions, which is an immediate consequence of Corollary 2.6.1 in [49].

Let $\phi :R_{\geq 0}^{n}\to R$ and let $e_{1},\ldots e_{n}$ denote the canonical basis of $R^{n}$. The function ϕ is supermodular if and only if, for all $r\in R_{+}^{n}$, all $\delta_{1},\delta_{2}\geq 0$ and all i,j with i≠j,

(3) $$\phi \left(r+\delta_{1}e_{i}+\delta_{2}e_{j}\right)+\phi \left(r\right)\geq \phi \left(r+\delta_{1}e_{i}\right)+\phi \left(r+\delta_{2}e_{j}\right).$$

Moreover, if ϕ has second partial derivatives, ϕ is supermodular if and only if, for all $r\in R_{+}^{n}$ and all i,j with i≠j,

(4) $$\frac{\partial^{2}\phi \left(r\right)}{\partial r_{i}\partial r_{j}}\geq 0.$$

The property characterized by Equations (3) and (4) is known in the economics literature as *increasing differences* [49]. This name is due to the effect an increase on a coordinate has on the value of ϕ being monotonically increasing with regard to the other coordinates. This is readily noticeable if we rearrange the terms of (3):

$$\phi \left(r+\delta_{1}e_{i}+\delta_{2}e_{j}\right)-\phi \left(r+\delta_{2}e_{j}\right)\geq \phi \left(r+\delta_{1}e_{i}\right)-\phi \left(r\right).$$

That is, the change of ϕ prompted by an increase of $\delta_{1}$ in coordinate *i* is greater the greater the value of coordinate *j*. Equation (3) is thus just the statement that on the lattice $\left(R_{\geq 0}^{n},⪯\right)$ increasing differences and supermodularity are equivalent concepts ([49] Corollary 2.6.1).

Using this result, as well as appealing directly to Definition 4, we now prove channel-supermodularity for a number of commonly used entropies. Throughout, given $r\in R_{\geq 0}^{n}$, we denote by $r_{i}$ its *i*th coordinate.

**Proposition** **2.**

*1.*  *Shannon entropy is channel-supermodular.*
*2.*  *Arimoto–Rényi entropies are channel-supermodular for all α∈(0,1)∪(1,∞).*
*3.*  *For any k, the k-tries entropy is channel-supermodular. In particular, min-entropy is channel-supermodular.*
*4.*  *Guessing entropy is channel-supermodular.*

**Proof.**  See Appendix A.    □

Items 3 and 4 of Proposition 2 are of particular interest to security applications, as guessing entropy and k-tries entropies have found interesting applications in the field of quantitative information flow.

Guessing entropy is especially useful in scenarios modelling brute-force attacks, as it models the expected number of attempts necessary for an adversary to obtain the value of a secret when trying one by one. On the other hand, *k*-tries entropy reflects the probability of guessing a value correctly when *k* guesses are allowed (see e.g., [19] (Section III.C)). It is defined as

$$H_{k-tries}\left(p\right)=-\log\sum_{i=1}^{k}p_{\left[i\right]},$$

and can be readily seen to be core-concave by taking η(x)=−log(−x) and $F\left(p\right)=-\sum_{i=1}^{k}p_{\left[i\right]}$. Notice that min entropy is equal to $H_{k-tries}$ when k=1.

The results in Proposition 2 justify our interest in channel-supermodularity, as any property derived for entropies in S will also hold for this set of commonly used entropy measures. However, not all entropies are channel-supermodular.

This includes another interesting entropy family useful in security, the *partition entropies* [19]. Let P be a partition of the set {1,…,n}. The partition entropy with regard to P is given by

$$H_{P}\left(X\right)=-\log\max_{A\in P}\sum_{i\in A}p\left(x_{i}\right).$$

It is easy to see that $H_{P}$ is core-concave, by taking η(x)=−log(−x) and $F\left(p\right)=-\max_{A\in P}\sum_{i\in A}p_{i}$. The partition entropy $H_{P}$ is useful for capturing the uncertainty of an *adversary* that is interested in knowing only to which subset the realization of *X* pertains. This is an appropriate model for adversaries that are interested in obtaining some specific partial knowledge about some sensitive information (e.g., obtaining the home town or the DOB of a user).

**Proposition** **3.**

*1.*  *Hayashi–Rényi, Tsallis and Sharma–Mittal entropies (with conditional forms as in Table 1) are not channel-supermodular for all α>1 whenever the input set is of size greater than 2. Moreover, they are also not channel-supermodular for all α∈(0,1) for some size of input set.*
*2.*  *The partition entropy is not, in general, channel-supermodular.*

**Proof.**  See Appendix A.    □

Notice that, for some choices of P, $H_{P}$ is channel-supermodular. In particular, if P={{i}|i∈{1,…,n}}, $H_{P}$ coincides with min-entropy.

A generalization of partition entropy is the *weighted partition entropies*,

$$H_{P,w}\left(X\right)=-\log\max_{A\in P}\sum_{i\in A}p\left(x_{i}\right)w_{i}$$

where $w=\left(w_{1},\ldots ,w_{n}\right)\in R_{\geq 0}^{n}$ is a set of weights. Being a generalization of partition entropies, weighted partition entropies are also not channel-supermodular in general.

## 4. The Join-Meet Operator and a New Structural Order

In this section we address the claim made in Section 1, proving that the *Join-Meet* operation is monotonic with regard to conditional entropy for all channel-supermodular entropies.

Let K:X→Y be a channel, with $Y=\{y_{1},\ldots ,y_{m}\}$, and let $K^{i}$ be the column of *K* corresponding to output $y_{i}$. Define, for i≠j, the *Join-Meet* operator ${⋄}_{i,j}$ as follows:

$$\begin{array}{c} {\left({⋄}_{i,j}K\right)}^{l}=\left\{\begin{array}{cc} K^{i}\vee K^{j} & \mathrm{if}l=i,\\ K^{i}\wedge K^{j} & \mathrm{if}l=j,\\ K^{l} & \mathrm{otherwise}. \end{array}\right. \end{array}$$

The next result proves that the Join-Meet operator is monotonic with $I_{H}$ if H∈S.

**Theorem** **4.** *For all channels $K_{1}$ and all i,j, $K_{1}\geq_{\mathrm{mc}}^{S}{⋄}_{i,j}K_{1}$.*

**Proof.**  Let H=(η,F)∈S and define $G_{F}$ as in Definition 5. Let $K_{2}={⋄}_{i,j}K_{1}$, and denote by $Y_{1}$, $Y_{2}$ the outputs of $K_{1}$, $K_{2}$. Notice that, for any distribution on the input, we have $p_{X,Y_{2}}\left(x_{k},y_{i}\right)=\max\left(p_{X,Y_{1}}\left(x_{k},y_{i}\right),p_{X,Y_{1}}\left(x_{k},y_{j}\right)\right)$, and, similarly, $p_{X,Y_{2}}\left(x_{k},y_{j}\right)=\min\left(p_{X,Y_{1}}\left(x_{k},y_{i}\right),p_{X,Y_{1}}\left(x_{k},y_{j}\right)\right)$. Thus,

$$\begin{array}{cc} \; & \sum_{l}G_{F}\left(p_{X,Y_{2}}\left(x_{1},y_{l}\right),\ldots ,p_{X,Y_{2}}\left(x_{n},y_{l}\right)\right)\\ \geq & \sum_{l=i,j}G_{F}\left(p_{X,Y_{1}}\left(x_{1},y_{l}\right),\ldots ,p_{X,Y_{1}}\left(x_{n},y_{l}\right)\right)\\ \; & +\sum_{l\neq i,j}G_{F}\left(p_{X,Y_{2}}\left(x_{1},y_{l}\right),\ldots ,p_{X,Y_{2}}\left(x_{n},y_{l}\right)\right)\\ = & \sum_{l}G_{F}\left(p_{X,Y_{1}}\left(x_{1},y_{l}\right),\ldots ,p_{X,Y_{1}}\left(x_{n},y_{l}\right)\right) \end{array}$$

where the inequality follows from $G_{F}$ being supermodular. From Equation (2) and η being increasing, it follows that $H\left(X|Y_{1}\right)\leq H\left(X|Y_{2}\right)$, which is equivalent to $I_{H}\left(X;Y_{1}\right)\geq I_{H}\left(X;Y_{2}\right)$.    □

In light of Theorem 4, one might wonder if the Join-Meet operator completely defines S—that is, whether H∈S whenever $K\geq_{\mathrm{mc}}^{H}{⋄}_{i,j}K$ for all channels *K* and all i,j. In fact, an even stronger statement can be made by only considering a subset of channels.

**Definition** **7.** *Let $K_{\left(k,l,\epsilon_{1},\epsilon_{2}\right)}$ denote the channel with input alphabet $\left\{x_{1},\ldots ,x_{n}\right\}$ and output alphabet $\left\{y_{1},y_{2}\right\}$, given by*

K(k,l,ϵ1,ϵ2)(y|x)=1−ϵ1ifx=xk,y=y1,ϵ1ifx=xk,y=y2,ϵ2ifx=xl,y=y1,1−ϵ2ifx=xl,y=y2,12otherwise.

**Theorem** **5.** *Let H=(η,F)∈H. If for all k,l≤n, and all ϵ1,ϵ2∈[0,12), $K_{\left(k,l,\epsilon_{1},\epsilon_{2}\right)}\geq_{\mathrm{mc}}^{H}{⋄}_{1,2}K_{\left(k,l,\epsilon_{1},\epsilon_{2}\right)}$, then H=(η,F)∈S.*

**Proof.**  We prove the contrapositive. Suppose that H=(η,F)∉S. Then, from (3), there are $r=\left(r_{1},\ldots ,r_{n}\right)\in R_{\geq 0}^{n}$, i,j≤n with i≠j and $\delta_{1},\delta_{2}>0$ such that

$$G_{F}\left(r+\delta_{1}e_{i}+\delta_{2}e_{j}\right)+G_{F}\left(r\right)<G_{F}\left(r+\delta_{1}e_{i}\right)+G_{F}\left(r+\delta_{2}e_{j}\right).$$

Let $\gamma ={\left(2{∥r∥}_{1}+\delta_{1}+\delta_{2}\right)}^{-1}$ and define a probability distribution over *X* by

$$p_{X}\left(x\right)=\left\{\begin{array}{cc} \gamma (2r_{i}+\delta_{1}), & \;\mathrm{if}\;x=x_{i},\\ \gamma (2r_{j}+\delta_{2}), & \;\mathrm{if}\;x=x_{j},\\ 2\gamma r_{l}, & \;\mathrm{if}\;x=x_{l},l\neq i,j. \end{array}\right.$$

Let $\epsilon_{1}=\frac{r_{i}}{\left(2r_{i}+\delta_{1}\right)}$ and $\epsilon_{2}=\frac{r_{j}}{\left(2r_{j}+\delta_{2}\right)}$, and let $K_{1}=K_{\left(i,j,\epsilon_{1},\epsilon_{2}\right)}$, $K_{2}={⋄}_{1,2}K_{1}$. Then,

$$\begin{array}{ccc} H(X|Y_{1}) & =\eta \left(\gamma \left(G_{F}\left(r+\delta_{1}e_{i}\right)+G_{F}\left(r+\delta_{2}e_{j}\right)\right)\right), & \;\mathrm{and}\\ H(X|Y_{2}) & =\eta \left(\gamma \left(G_{F}\left(r+\delta_{1}e_{i}+\delta_{2}e_{j}\right)+G_{F}\left(r\right)\right)\right), \end{array}$$

and thus, as η is strictly increasing, $H\left(X|Y_{1}\right)>H\left(X|Y_{2}\right)$, which concludes the proof.    □

An immediate consequence of Theorems 4 and 5 is that the Join-Meet operator completely characterizes S.

**Corollary** **1.** *Let H∈H. H∈S if, and only if, $K\geq_{\mathrm{mc}}^{H}{⋄}_{i,j}K$ for all channels K and all i,j.*

### A New Structural Ordering

Theorem 4 yields some immediate new results for reasoning about channel ordering, as, whenever |X|>2, the Join-Meet operator is not, in general, captured by the degradedness ordering. Consider, for instance, the following channels $K_{1}$,$K_{2}$ (notice that $K_{1}=K_{\left(1,2,0,0\right)}$).

(5) 1001121210101212

Then, we have that $K_{2}={⋄}_{1,2}K_{1}$, but clearly $K_{1}{≱}_{d}K_{2}$. To see this, fix a channel $R:Y_{1}\to Y_{2}$. Because *R* is a channel, there are p,q∈[0,1] such that

$$R=\left(\begin{array}{cc} p & 1-p\\ q & 1-q \end{array}\right).$$

Then, we have

K1R=p1−pq1−q(12)(p+q)(12)(2−p−q).

Therefore, $K_{2}\neq K_{1}R$ for any choice of p,q.

We formalize this observation in the next result.

**Proposition** **4.**

*1.*  *If |X|=2, then, for all K:X→Y and all i,j≤|Y|, $K\geq_{d}{⋄}_{i,j}K$.*
*2.*  *If |X|>2, then there are K:X→Y and i,j≤|Y| such that $K{≱}_{d}{⋄}_{i,j}K$.*

**Proof.**  We first prove (1). As it is possible to reorder columns by degrading a channel, without loss of generality let i=1 and j=2. Fix K:X→Y. Let $K_{kl}=K\left(y_{l}|x_{k}\right)$, and suppose, again without loss of generality, that $K_{11}\geq K_{12}$ and $K_{22}\geq K_{21}$.

If $K_{11}=K_{12}$ or $K_{22}=K_{21}$, then ${⋄}_{1,2}K$ is obtainable by permutating columns of *K*, and therefore $K\geq_{d}{⋄}_{1,2}K$. Otherwise, we have $K_{11}K_{22}-K_{12}K_{21}>0$, and ${⋄}_{1,2}K=KR$ where *R* is the following channel:

$$\begin{array}{cccccc} R & y_{1} & y_{2} & y_{3} & \cdots & y_{m}\\ y_{1} & \frac{K_{22}K_{11}-K_{22}K_{12}}{K_{11}K_{22}-K_{12}K_{21}} & \frac{K_{22}K_{12}-K_{12}K_{21}}{K_{11}K_{22}-K_{12}K_{21}} & 0 & \cdots & 0\\ y_{2} & \frac{K_{11}K_{22}-K_{11}K_{21}}{K_{11}K_{22}-K_{12}K_{21}} & \frac{K_{11}K_{21}-K_{12}K_{21}}{K_{11}K_{22}-K_{12}K_{21}} & 0 & \cdots & 0\\ y_{3} & 0 & 0 & 1 & \cdots & 0\\ \vdots & \vdots & \vdots & \vdots & \ddots & \vdots \\ y_{m} & 0 & 0 & 0 & \cdots & 1 \end{array}$$

For the proof of (2), it suffices to notice that, whenever |X|>2, $K_{\left(1,2,0,0\right)}{≱}_{d}{⋄}_{1,2}K_{\left(1,2,0,0\right)}$. The proof for general |X| is along the same lines of the argument after (5).    □

Next we define the channel-supermodularity preorder over channels, which is based on the Join-Meet operators ${⋄}_{i,j}$.

**Definition** **8.** *$K_{1}\geq_{s}K_{2}$ if there is a finite collection of tuples $\left(i_{k},j_{k}\right)$ such that*

$$K_{2}={⋄}_{i_{1},j_{1}}\left({⋄}_{i_{2},j_{2}}\left(\ldots {⋄}_{i_{m},j_{m}}K_{1}\right)\right).$$

An induced preorder can be then defined by combining $\geq_{d}$ and $\geq_{s}$ as follows:

**Definition** **9.** *$K_{1}\geq_{\mathrm{ds}}K_{2}$ if there are channels $W_{1},\ldots ,W_{n}$ such that $K_{1}\geq_{0}W_{1}\geq_{1}\ldots \geq_{n-1}W_{n}\geq_{n}K_{2}$, where each $\geq_{i}$ stands for $\geq_{d}$ or $\geq_{s}$.*

## 5. Relations between Preorders for Channel-Supermodular Entropies

Throughout this section, let $K_{1}:X\to Y$ and $K_{2}:X\to Z$. First, note that Proposition 1 and Theorem 2 are still meaningful under S. The next proposition summarizes the relationship between $\left(\geq_{\mathrm{ds}}\right)$ and the other preorders.

**Proposition** **5.**

*1.*  *$K_{1}\geq_{d}K_{2}\Rightarrow K_{1}\geq_{\mathrm{ds}}K_{2}$ and $K_{1}\geq_{s}K_{2}\Rightarrow K_{1}\geq_{\mathrm{ds}}K_{2}$,*
*2.*  *$K_{1}\geq_{\mathrm{ds}}K_{2}⇏K_{1}\geq_{d}K_{2}$,*
*3.*  *$K_{1}\geq_{\mathrm{ds}}K_{2}⇏K_{1}\geq_{s}K_{2}$,*
*4.*  *$K_{1}\geq_{\mathrm{ds}}K_{2}⇏K_{1}\geq_{\ln}^{S}K_{2}$,*
*5.*  *$K_{1}\geq_{\ln}^{S}K_{2}\Rightarrow K_{1}\geq_{\mathrm{ds}}K_{2}$,*
*7.*  *$K_{1}\geq_{\ln}^{H_{1}}K_{2}⇏K_{1}\geq_{\mathrm{ds}}K_{2}$,*
*8.*  *$K_{1}\geq_{\mathrm{ds}}K_{2}\Rightarrow K_{1}\geq_{\mathrm{mc}}^{S}K_{2}$,*
*8.*  *$K_{1}\geq_{\mathrm{ds}}K_{2}⇏K_{1}\geq_{\mathrm{sh}}K_{2}$,*
*9.*  *$K_{1}\geq_{\mathrm{sh}}K_{2}⇏K_{1}\geq_{\mathrm{ds}}K_{2}$.*

**Proof.**  See Appendix B.    □

Proposition 5.7 can be used to decide whether $K_{1}\geq_{\mathrm{mc}}^{H}K_{2}$, for H∈S, by only using structural properties of the channel. Consider, for example, the following channels $K_{1}$, $K_{2}$.

120120121212120143414343525.

In [19], the authors claimed to have no proof that $K_{1}\geq_{\mathrm{mc}}^{H_{1}}K_{2}$. By Proposition 5 (7), $K_{1}\geq_{\mathrm{mc}}^{H_{1}}K_{2}$ can be proven as follows:

120120121212120≥s120121201212120≥d143414343525.

Notice that if H is substituted for S, Theorem 3 does not hold:

**Proposition** **6.** *$K_{1}\geq_{\mathrm{mc}}^{S}K_{2}⇏K_{1}\geq_{d}K_{2}$ and $K_{1}\geq_{\mathrm{mc}}^{S}K_{2}⇏K_{1}\geq_{\ln}^{S}K_{2}$.*

**Proof.**  From Proposition 5 (2), there are channels $K_{1}$,$K_{2}$ such that $K_{1}\geq_{\mathrm{ds}}K_{2}$ and $K_{1}{≱}_{d}K_{2}$. For such channels, Proposition 5 (7) implies $K_{1}\geq_{\mathrm{mc}}^{S}K_{2}$, and the first result follows. The second result then follows by noting that Proposition 1 and Theorem 2 imply $K_{1}\geq_{\ln}^{S}K_{2}\Leftrightarrow K_{1}\geq_{d}K_{2}$.    □

**Proposition** **7.** *$K_{1}\geq_{\mathrm{mc}}^{S}K_{2}⇏K_{1}\geq_{\mathrm{sh}}K_{2}$.*

### Results on Channel Capacity

In [1], Shannon proved that $K_{1}\geq_{\mathrm{sh}}K_{2}\Rightarrow K_{1}\geq_{c}^{H_{1}}K_{2}$. We can use Theorem 4 to prove similar results for the preorder $\geq_{\mathrm{shs}}$, which is an extension of $\geq_{\mathrm{sh}}$ with $\geq_{s}$.

**Definition** **10.** *$K_{1}\geq_{\mathrm{shs}}K_{2}$ if there are channels $W_{1},\ldots ,W_{n}$ such that $K_{1}\geq_{0}W_{1}\geq_{1}\ldots \geq_{n-1}W_{n}\geq_{n}K_{2}$ where each $\geq_{i}$ stands for $\geq_{\mathrm{sh}}$ or $\geq_{s}$.*

We have:

**Proposition** **8.** *For all channels $K_{1},K_{2}$,*

*1.*  *$K_{1}\geq_{\mathrm{sh}}K_{2}\Rightarrow K_{1}\geq_{\mathrm{shs}}K_{2}$,*
*2.*  *$K_{1}\geq_{\mathrm{ds}}K_{2}\Rightarrow K_{1}\geq_{\mathrm{shs}}K_{2}$,*
*3.*  *$K_{1}\geq_{\mathrm{shs}}K_{2}⇏K_{1}\geq_{s}K_{2}$,*
*4.*  *$K_{1}\geq_{\mathrm{shs}}K_{2}⇏K_{1}\geq_{\mathrm{sh}}K_{2}$.*

**Proof.**  (1) and (2) follow immediately from the definitions of $\geq_{\mathrm{ds}}$ and $\geq_{\mathrm{shs}}$, and from observing that $\geq_{d}\Rightarrow \geq_{\mathrm{sh}}$. (3) and (4) follow from (2) and Propositions 5 (3) and 5 (8).    □

In the remainder of this section, we prove that $K_{1}\geq_{\mathrm{shs}}K_{2}$ is a sufficient condition for establishing that both the Shannon and min-capacity of $K_{1}$ are at least as large as that of $K_{2}$.

**Lemma** **1.** *Let H∈S. If $K_{1}\geq_{\mathrm{sh}}K_{2}\Rightarrow K_{1}\geq_{c}^{H}K_{2}$, then $K_{1}\geq_{\mathrm{shs}}K_{2}\Rightarrow K_{1}\geq_{c}^{H}K_{2}$.*

**Proof.**  Let H∈S. From Propositions 1, 5 (1), and 5 (7), $K_{1}\geq_{s}K_{2}\Rightarrow K_{1}\geq_{c}^{H}K_{2}$. The result then follows from Definition 10.    □

**Proposition** **9.**

*1.*  *$K_{1}\geq_{\mathrm{shs}}K_{2}\Rightarrow K_{1}\geq_{c}^{H_{1}}K_{2}$,*
*2.*  *$K_{1}\geq_{\mathrm{shs}}K_{2}\Rightarrow K_{1}\geq_{c}^{H_{\infty }}K_{2}$,*
*3.*  *$K_{1}\geq_{c}^{H_{1}}K_{2}⇏K_{1}\geq_{\mathrm{shs}}K_{2}$ and $K_{1}\geq_{c}^{H_{\infty }}K_{2}⇏K_{1}\geq_{\mathrm{shs}}K_{2}$.*

**Proof.**  See Appendix B.    □

**Figure 1:**
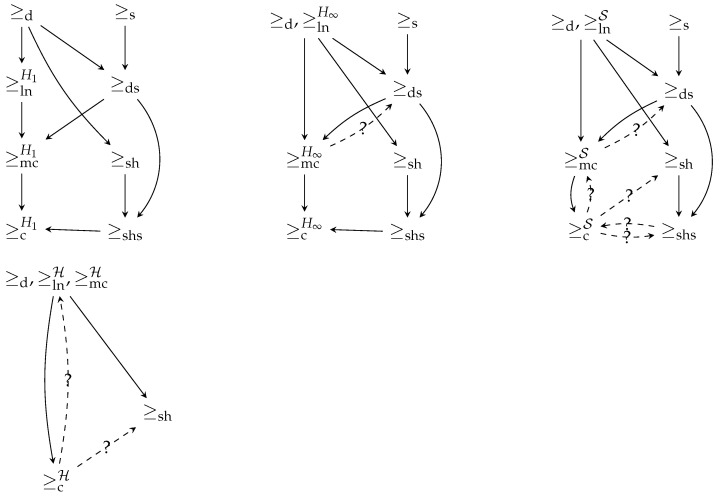
The four implication graphs summarize the relation between preorders. It is known that a preorder $\geq_{i}$ implies a preorder $\geq_{j}$ if and only if there is a path of solid arrows from $\geq_{i}$ to $\geq_{j}$. Preorders that are equivalent are grouped together, and the dotted arrows represent an implication whose validity is an open question. When no path is present, the implication is known not to hold.

Figure 1 summarizes the implications between orderings. As can be seen, there are some open questions that need to be established, designated by dotted lines with a question mark. Note that the absence of an arrow means that the implication is known to be false.

## 6. Channel Design

Core-concavity was originally introduced in [51] in the context of universally optimal channel design—that is, the problem of finding, given some operational constraints, a channel leaking the minimum amount of confidential information (optimality), for all entropy-based measures of leakage (universality). This section shows how core-concavity and channel-supermodularity can be used in this context.

If *X* and *Y* are the input and output of a channel and *H* is a core-concave entropy, then the leakage about *X* through *Y* as measured by *H* is defined to be the *H*-mutual information $I_{H}\left(X;Y\right)$, as in Definition 3.

The concept of leakage is relevant in security/privacy contexts where *X* is some confidential data, *K* is modelling a system (e.g., a cryptographic computation or a database query system), and *Y* some observable generated by the system (e.g., the computation time or the result of a statistical query). Different *H* corresponds to different attackers’ models, and universal channel solutions identify countermeasures to leakage which are robust with regard to all attackers in that universe.

Minimizing leakage of sensitive information is usually a desirable goal. However, when designing systems, it is often the case that some leakage is unavoidable. With that in mind, some recent works in QIF aimed at obtaining channels that leak the least amount of information subject to some operational constraints [13,28,29]. From Definition 3, the problem can be rephrased as finding the channel which, subject to some operational constraints, maximizes H(X|Y) — or, as η is increasing, maximizes $\sum_{y}p\left(y\right)F\left(X|y\right)$. In a recent work [29], which considered a generalizing framework for these operational constraints, it was shown that this problem can be solved by convex optimization techniques, for a given core-concave *H*. However, it was also proven that the solution to the problem is in general not *universal*—that is, the optimal channel given a set of constraints may vary with the choice of *H*.

Despite this negative result, it was shown in [29] that some classes of problems admit a universal solution. As different entropies model different attackers, these results provide a very strong security guarantee—namely, that the optimal system in these situations is the most secure possible regardless of the attacker model. In the next few sections, we show how channel-supermodularity can be a useful tool in obtaining solutions that, while not universally optimal for all core-concave entropies, are the most secure for all symmetric entropies in S.

### 6.1. Deterministic Channel Design Problem: A Universal Solution by Channel-Supermodularity

In many applications, such as repeated queries, it is either undesirable or impractical to consider a “probabilistic” system. This motivates the study of the channel design problem restricted to *deterministic* channels, which has been recently investigated in [13].

It was proven in [13] that, similarly to the general channel design problem, the deterministic version does not in general accept a universal solution. Moreover, the problem was also shown to be, in general, NP-hard. However, it was also proven in this work that a specific class of problems, called the *deterministic complete k-hypergraph design problem*, admit a solution that is optimal for all symmetric channel-supermodular entropies. This problem can be defined as follows.

**Definition** **11.** *Let H∈H, $k\in N_{>0}$ and let X,Y be finite sets with |Y|≥|X|/k*

*The deterministic complete k-hypergraph design problem (CKDP) is to find a channel K:X→Y that maximizes H(X|Y), subject to the following constraints:*

- *∀x,y,K(y|x)∈{0,1}, and*
- *$\forall y,\;\;\;\;\sum_{x}K\left(y|x\right)\leq k$.*

That is, the deterministic CKDP is the problem of finding the most secure deterministic channel, subject to the constraint that each output can only be generated by at most *k* inputs.

For the remainder of this section, let $k\in N_{>0}$, and fix a distribution $p_{X}$ such that, without loss of generality, $p_{X}\left(x_{i}\right)\geq p_{X}\left(x_{j}\right)$ whenever i<j.

**Figure 2:**
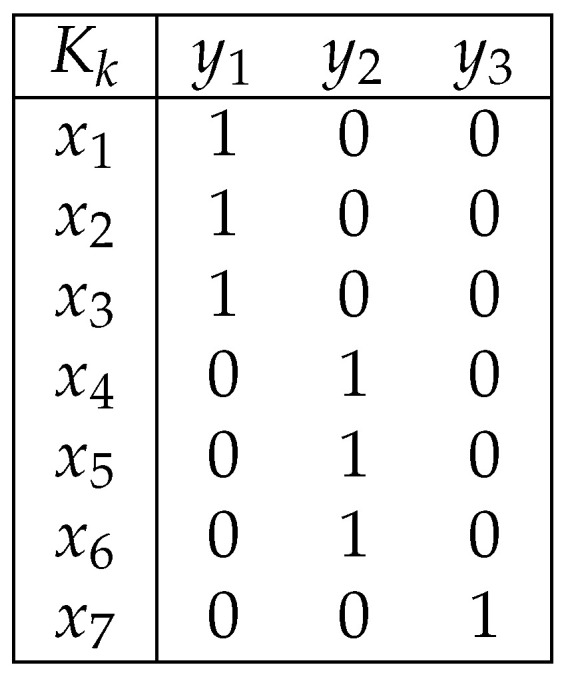
The solution given by Algorithm 1 for k=3.

The greedy solution proposed by [13] is described in Algorithm 1. The algorithm is straightforward: it associates the *k* most likely secrets with the first observable; it then associates the *k* most likely secrets among the remaining secrets with the second observable, and so on. The solution for $X=\{x_{1},\ldots ,x_{8}\}$ and k=3 is depicted in Figure 2.
**Algorithm 1** Greedy algorithm for the *k*-complete hypergraph problem**Input:** Input set X, prior $p_{X}$ and integer k≤|X|
**Output:** Matrix of optimal deterministic channel $K_{k}$  1: **initialize:**
$K_{k}$ as a matrix of 0s, with |X| rows and ⌈|X|/k⌉ columns.  2: **for**
i∈{1,…,|X|}
**do**  3:    $K_{k}\left(i,\left\lceil i\;/\;k\right\rceil \right)=1$  4:**return**
$K_{k}$

**Theorem** **6** ([13]). *Given a complete k-hypergraph channel design problem, the solution given by Algorithm 1 is optimal for any symmetric channel-supermodular entropy.*

**Proof.**  We reproduce the proof of this theorem from [13], as it provides an interesting application of channel-supermodularity. Let us consider the following joint matrix $J_{k}$ obtained by Algorithm 1 and the prior:

$$\begin{array}{c} \left(\begin{array}{cccc} p_{1} & 0 & \ldots & 0\\ \vdots & \vdots & & \vdots \\ p_{k} & 0 & & \vdots \\ 0 & p_{k+1} & & \vdots \\ \vdots & \vdots & & \vdots \\ \vdots & p_{2k} & & \vdots \\ \vdots & 0 & & \vdots \\ \vdots & \vdots & \ddots & \vdots \\ \;\vdots & \vdots & & 0\\ \vdots & \vdots & & p_{mk+1}\\ \;\vdots & \vdots & & \vdots \\ 0 & 0 & \ldots & p_{n} \end{array}\right) \end{array}$$

We now prove that any matrix *J* satisfying the constraints can be transformed into $J_{k}$ by a sequence of steps each increasing (or keeping equal) any supermodular entropy. Each step consists of the following three sub-steps:

- Select two columns $c_{i},c_{j}$ and align the non-zero coefficients in $c_{i},c_{j}$;
- Perform ∧,∨ operations on the aligned columns and replace $c_{i},c_{j}$ with $c_{i}\vee c_{j},c_{i}\wedge c_{j}$;
- Disalign the two columns $c_{i}\vee c_{j},c_{i}\wedge c_{j}$.

The following example illustrates one step (i.e., the three sub-steps above):

$$\left(\begin{array}{ccc} 0 & 0.4 & 0\\ 0 & 0 & 0.3\\ 0 & 0.15 & 0\\ 0 & 0 & 0.1\\ 0.05 & 0 & 0 \end{array}\right)\overset{=}{\to }\left(\begin{array}{ccc} 0 & 0.4 & 0.1\\ 0 & 0.15 & 0.3\\ 0 & 0 & 0\\ 0 & 0 & 0\\ 0.05 & 0 & 0 \end{array}\right)\overset{\leq }{\to }\left(\begin{array}{ccc} 0 & 0.4 & 0.1\\ 0 & 0.3 & 0.15\\ 0 & 0 & 0\\ 0 & 0 & 0\\ 0.05 & 0 & 0 \end{array}\right)\overset{=}{\to }\left(\begin{array}{ccc} 0 & 0.4 & 0\\ 0 & 0.3 & 0\\ 0 & 0 & 0.15\\ 0 & 0 & 0.1\\ 0.05 & 0 & 0 \end{array}\right)$$

from left to right we have:

- selected $c_{2},c_{3}$, which are the two columns containing the two most likely priors (i.e., 0.4 and 0.3) and aligned $c_{2},c_{3}$ so that 0.4, 0.3 appear in different rows;
- replaced $c_{2},c_{3}$ with $c_{2}\vee c_{3},c_{2}\wedge c_{3}$;
- disaligned columns 2 and 3, that is, position values in $c_{2}\vee c_{3},c_{2}\wedge c_{3}$, so that each row has the same probability it had before the step.

Notice that aligning (and disaligning) is a permutation of a column; hence, they do not change the value of the posterior of symmetric channel-supermodular entropies because $G_{F}\left(c_{i}\right)=G_{F}\left(c_{i}^{\prime }\right)$ for any permutation $c_{i}^{\prime }$ of the column $c_{i}$.

Next, for the remaining sub-step, where we replace $c_{i},c_{j}$ with $c_{i}\vee c_{j},c_{i}\wedge c_{j}$, by supermodularity of *G* we have $G_{F}\left(c_{i}\right)+G_{F}\left(c_{j}\right)\leq G_{F}\left(c_{i}\vee c_{j}\right)+G_{F}\left(c_{i}\wedge c_{j}\right)$; hence, that sub-step increases (or keeps equal) the posterior entropy. Notice also that the matrix at the end of the step has in each row the same probabilities as it had before that step; hence, it is still a joint matrix that respects the complete *k*-hypergraph constraints for the same prior.

The selection and alignment of columns is as follows: at the initial step select $c_{i}$ such that $c_{i}$ contains the first *r* elements with the highest probabilities, say $p_{1},\ldots ,p_{r}$; if r<k, then select $c_{j}$ as the column containing $p_{r+1}$; align $c_{i},c_{j}$ so that $p_{r+1}$ is not on the same row as any of the $p_{1},\ldots ,p_{r}$ (and $c_{i}\vee c_{j}$ has no more than *k* non-zero terms). Then, $c_{i}\vee c_{j}$ will contain $p_{1},\ldots ,p_{r+1}$. Repeat until r=k. Then repeat the process considering the probabilities $p_{k+1},\ldots ,p_{n}$.

By repeating these steps, we will reach a matrix $J^{\prime }$ with columns $c_{1}^{\prime },\ldots ,c_{n}^{\prime }$ such that each element of column $c_{i}^{\prime }$ has higher probability than all elements of column $c_{i+1}^{\prime }$. This is exactly the solution given by the greedy algorithm (modulo column permutations), that is, $J^{\prime }=J_{k}$. □

If *H* is not channel-supermodular, the greedy solution may not be optimal. Consider, for example, the Hayashi–Rényi entropies, which are not channel-supermodular. Let $X=\{x_{1},\ldots ,x_{4}\}$, $p_{X}=\left(0.3,0.3,0.2,0.2\right)$ and k=3, and consider the channels $K_{1},K_{2}$ with outputs $Y_{1},Y_{2}$ below.

$$\begin{array}{ccc} K_{1} & y_{1} & y_{2}\\ x_{1} & 1 & 0\\ x_{2} & 1 & 0\\ x_{3} & 1 & 0\\ x_{4} & 0 & 1 \end{array}\;\begin{array}{ccc} K_{2} & y_{1} & y_{2}\\ x_{1} & 1 & 0\\ x_{2} & 1 & 0\\ x_{3} & 0 & 1\\ x_{4} & 0 & 1 \end{array}$$

Then, $K_{1}$ is the greedy solution. However, for Hayashi–Rényi entropies, the following limit holds [52]:

$$\lim_{\alpha \to \infty }H_{\alpha }^{\prime }\left(X|Y\right)=-\log\left(\max_{y\in ∼≊∥(Y),\;x\in X}p_{X|y}\left(x\right)\right),$$

Therefore,

$$\lim_{\alpha \to \infty }H_{\alpha }^{\prime }\left(X|Y_{1}\right)=0,$$

whereas

$$\lim_{\alpha \to \infty }H_{\alpha }^{\prime }\left(X|Y_{2}\right)=1.$$

Thus, $H_{\alpha }^{\prime }\left(X|Y_{1}\right)<H_{\alpha }^{\prime }\left(X|Y_{2}\right)$ for large enough α, and the greedy solution is not optimal.

Another example of core-concave functions for which Algorithm 1 is not optimal is provided by “partition” entropies. For example, if *B* is a partition of the possible values of *X* then

$$H_{B}\left(X\right)=-\log\max_{S\in B}\sum_{x\in S}p_{X}\left(x\right)$$

is a core-concave entropy which is not channel-supermodular.

### 6.2. An Application to Query Anonymity

Let us consider the following anonymity mechanism problem: we want to design an anonymity mechanism where in order to conceal a secret query from an eavesdropper, the user sends to a server a set of *k* queries which includes the secret query. Then, once received from the server the response to all the *k* queries, the user retrieves the response to the secret query. In our setting, this corresponds to each observable having a pre-image of size exactly *k*.

As an illustrative example consider a Twitter user who wants to visit some other Twitter user page but wants to keep this query secret. To solve this problem, he decides to use the following protocol: whenever he visits the desired user page, he also sends k−1 other queries to the pages of other Twitter users. Suppose further that this user frequently visits this user’s page, meaning that a random choice of the other queries is not a wise strategy, since multiple observations would end up revealing more and more information about the query, eventually completely revealing the secret query. The problem is then: which set of *k* Twitter pages will leak the least information about the user secret query?

We assume the attacker has no background information about the user, and hence we set the probability of a Twitter query for that user as the probability that a general member of the public requests that Twitter page (a good proxy to this measure can be derived by the number of followers of that Twitter page). Let *n* be the number of possible queries (i.e., the input set). Considering the scenario that *n* is divisible by *k*, we can use Algorithm 1 to solve this problem.

Notice that for *n* secrets, there are $n!{\left(\frac{n}{k}!\right)}^{-1}{\left(k!\right)}^{-\frac{n}{k}}$ possible ways to satisfy these anonymity constraints. For example, there are about $7\times 10^{85}$ possible solutions when n=100 and k=10, and about $4\times 10^{19,704}$ for n= 10,000 and k=100. We will now compare the greedy algorithm in Section 6 against other possible anonymity solutions, and we will measure the goodness of the solutions using min and Shannon posterior entropies. Let us consider the three anonymity solutions below:

1. the solution from the greedy algorithm (Algorithm 1) (i.e., pick the k−1 queries closest in probability to the real query);
2. a random solution (i.e., pick *k* random queries);
3. a non-optimal solution where the secrets with the highest probabilities, instead of being grouped in the first bin, are distributed in the other bins.

For example, for 6 secrets and k=2, the greedy solution would be $\{\left\{x_{1},x_{2}\right\},$$\{x_{3},x_{4}\},$$\left\{x_{5},x_{6}\}\right\}$, whereas the non-optimal solution (3) would be $\{\left\{x_{1},x_{4}\right\},$$\{x_{2},x_{5}\},$$\left\{x_{3},x_{6}\}\right\}$.

**Figure 3:**
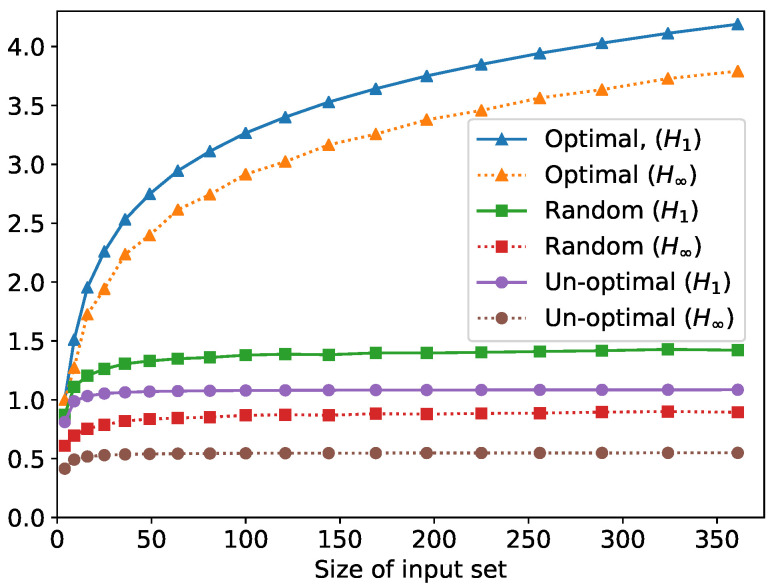
Posterior Shannon and min-entropy for different anonymity solutions. *k* is $\sqrt{n}$, where *n* is the size of the input set. For the random solution, the values in the plot are the average of 1000 samples.

The difference between these solutions can be very substantial. Figure 3 shows the values when the distribution over the input set is a binomial distribution, with parameter p=0.5: in this scenario, supposing that there is a universe of 350 Twitter pages and that the user sends 19 queries selected using the greedy algorithm, the probability of an attacker guessing the secret query correctly would be over 7 times smaller than if the user had opted instead for the non-optimal solution (using $2^{-H_{\infty }\left(X|Y\right)}$ as the conversion formula from posterior min entropy to probability of guessing). In fact, it is easy to define an input probability distribution such that the leakage gap between the non-optimal solution and the optimal solution given by the greedy algorithm is arbitrarily large.

Note that, by Theorem 6, the greedy solution is in fact optimal for *all* channel-supermodular entropies. Hence, the user knows that the greedy solution is optimal against an attacker trying to guess his secret query in a fixed number of guesses, or using guesswork or guessing using a twenty-questions-style guesswork (reflecting Shannon entropy), and so on.

### 6.3. Query Anonymity for Related Secrets

Consider the scenario where a user who wants to query the Twitter page of some political commentators is at the same time interested in hiding his own political affiliation, which could be leaked by his queries. In this scenario, the solution from Algorithm 1 might be sub-optimal. To see this, suppose that the *k* queries in the real query’s cover end up being all affiliated to the same party. This would thus reveal the user’s political party to the attacker with certainty even though the real intended query is still uncertain. This is not a contradiction to the optimality of the algorithm. As such, an adversary would be better modeled by a partition entropy—with political commentators (the queried users) grouped by party affiliation—and, as established in Proposition 3, this type of entropy is not channel-supermodular in general.

Motivated by this scenario, we now give an optimal solution for all channel supermodular entropies (even if not symmetric) to deal with this kind of problem. Suppose there are *k* political parties, and *l* commentators aligned with each party. Suppose, further, that the user is affiliated to one of the parties, and would like to check the profiles of his party’s commentators without revealing his own affiliation.

To achieve this aim, the user decides to group the political commentators in covers of size *k*, each cover containing exactly one commentator from each party, and then proceed to use these covers similarly to the mechanism described in Section 6.2, by querying the entire cover (fetching the pages of all the commentators in the cover). The question is: what is the set of covers that reveal the least amount of information about the user’s political affiliation?

Let $X=\{P_{1},\ldots P_{k}\}$ be the set of parties, $Y=\{c_{1}^{1},\ldots ,c_{1}^{l},c_{2}^{1},\ldots ,c_{2}^{l},\ldots ,\ldots ,c_{k}^{1},\ldots ,c_{k}^{l}\}$ be the set of commentators, wherein $c_{i}^{j}$ is the *j*th commentator of the *i*th party, and let $Z\subset 2^{Y}$ be the set of covers. Let K:X→Y be the channel giving the conditional probability of a user choosing to query a commentator given the user’s party inclination. We assume that the user only chooses commentators that share the same affiliation as his, that is, $K(c_{j}^{i}|P_{m})=0$ whenever j≠m. For simplicity, we assume that the commentators are organized decreasingly with regard to this probability—that is,

(6) $$K\left(c_{j}^{i}|P_{j}\right)\geq K\left(c_{j}^{m}|P_{j}\right)\;\mathrm{whenever}\;m>i.$$

We claim that the optimal mechanism, for all channel-supermodular entropies (with regards to the political parties, not the commentators), is to group the most popular commentators for each party in the first cover, then the second-most popular commentators for each party on the second, and so on. That is, the covers would be: $\{\left\{c_{1}^{1},c_{2}^{1},\ldots ,c_{k}^{1}\right\}$, $\left\{c_{1}^{2},c_{2}^{2},\ldots ,c_{k}^{2}\right\}$, *…*, $\left\{c_{1}^{l},c_{2}^{l},\ldots ,c_{k}^{l}\}\right\}$.

Let R:Y→Z be the channel mapping each commentator to their cover, modelling the optimal solution described above. The matrix of this channel can be seen as a vertical concatenation of identity matrices:

(7) $$\begin{array}{ccccc} R & Cover_{1} & Cover_{2} & \ldots & Cover_{l}\\ c_{1}^{1} & 1 & 0 & \ldots & 0\\ c_{1}^{2} & 0 & 1 & \ldots & 0\\ \vdots & \vdots & \vdots & \ddots & \vdots \\ c_{1}^{l} & 0 & 0 & \ldots & 1\\ \vdots & \; & \vdots & \; & \\ \vdots & \; & \vdots & \; & \\ c_{k}^{1} & 1 & 0 & \ldots & 0\\ c_{k}^{2} & 0 & 1 & \ldots & 0\\ \ldots & \ldots & \ldots & \ddots & \ldots \\ c_{k}^{1} & 0 & 0 & \ldots & 1 \end{array}\;\begin{array}{ccccc} KR & Cover_{1} & Cover_{2} & \ldots & Cover_{l}\\ P_{1} & K(c_{1}^{1}|P_{1}) & K(c_{1}^{2}|P_{1}) & \ldots & K(c_{1}^{l}|P_{1})\\ P_{2} & K(c_{2}^{1}|P_{2}) & K(c_{2}^{2}|P_{2}) & \ldots & K(c_{2}^{l}|P_{2})\\ \vdots & \vdots & \vdots & \; & \vdots \\ P_{k} & K(c_{k}^{1}|P_{k}) & K(c_{k}^{2}|P_{k}) & \ldots & K(c_{k}^{l}|P_{k}) \end{array}$$

The channel KR:X→Z above is then obtained by postprocessing *K* by *R*.

The claim of optimally can be more formally stated as follows: given any channel-supermodular *H*, any distribution $p_{X}$ over the parties, and any other deterministic covering $R^{\prime }:Y\to Z^{\prime }$ in which there is exactly one commentator from each party per cover, the resulting channel $KR^{\prime }$ will never leak less information than the channel KR in (7). That is,

$$H\left(X|Z\right)\geq H\left(X|Z^{\prime }\right).$$

The proof that the covering *R* above is indeed optimal for all channel-supermodular entropies is similar to the proof of Theorem 6, but even simpler. Suppose the channel $R^{\prime }:Y\to Z$ is any covering that satisfies the restriction that each cover has exactly one commentator from each party. Now, consider the channel $KR^{\prime }$, and proceed as follows: first, do the Join-Meet operation of the first column with all the other columns (that is, obtaining the channel ${⋄}_{1,l}{⋄}_{1,l-1}\ldots {⋄}_{1,2}\left(KR^{\prime }\right)$ ). Now, disregarding the first column, the process is repeated for the second column with all the remaining ones. It is easy to see that the resulting channel will be exactly KR in (7).

As an example, let k=3, l=3, and suppose the non-zero values of *K* are:

$$\begin{array}{c} K\left(c_{1}^{1}|P_{1}\right)=0.5,\;\;K\left(c_{1}^{2}|P_{1}\right)=0.3,\;\;K\left(c_{1}^{3}|P_{1}\right)=0.2\\ K\left(c_{2}^{1}|P_{2}\right)=0.6,\;\;K\left(c_{2}^{2}|P_{2}\right)=0.3,\;\;K\left(c_{2}^{3}|P_{2}\right)=0.1\\ K\left(c_{3}^{1}|P_{3}\right)=0.5,\;\;K\left(c_{3}^{2}|P_{3}\right)=0.4,\;\;K\left(c_{3}^{3}|P_{3}\right)=0.1 \end{array}$$

and suppose that the covers that are defined by $R^{\prime }$ are $\left\{c_{1}^{2},c_{2}^{1},c_{3}^{2}\right\}$, $\left\{c_{1}^{1},c_{2}^{3},c_{3}^{1}\right\}$, and $\left\{c_{1}^{3},c_{2}^{2},c_{3}^{3}\right\}$. We have

$$\begin{array}{cccc} KR^{\prime } & Cover_{1} & Cover_{2} & Cover_{3}\\ P_{1} & 0.3 & 0.5 & 0.2\\ P_{2} & 0.6 & 0.1 & 0.3\\ P_{3} & 0.4 & 0.5 & 0.1 \end{array}$$

By doing the Join-Meet operations on the first column with the others, we obtain

$$\begin{array}{cccc} {⋄}_{1,3}{⋄}_{1,2}\left(KR^{\prime }\right) & Cover_{1} & Cover_{2} & Cover_{3}\\ P_{1} & 0.5 & 0.3 & 0.2\\ P_{2} & 0.6 & 0.1 & 0.3\\ P_{3} & 0.5 & 0.4 & 0.1 \end{array}$$

Finally, by doing the Join-Meet of the second column with the remaining one, we have

$$\begin{array}{cccc} {⋄}_{2,3}{⋄}_{1,3}{⋄}_{1,2}\left(KR^{\prime }\right) & Cover_{1} & Cover_{2} & Cover_{3}\\ P_{1} & 0.5 & 0.3 & 0.2\\ P_{2} & 0.6 & 0.3 & 0.1\\ P_{3} & 0.5 & 0.4 & 0.1 \end{array}$$

which is exactly the optimal solution given by (7).

## 7. Conclusions

In this work, we introduced the notion of channel-supermodular entropies, as a subset of core-concave entropies, which include guessing and Arimoto–Rényi entropies. We demonstrated that, for this new classification of entropies, the Join-Meet operator on channel columns decreases the *H*-mutual information. This property prompted us to define structural preorders of channels ($\geq_{\mathrm{ds}},\geq_{\mathrm{sds}}$), providing novel sufficient conditions for establishing whether two channels are in the *H*-more capable any channel-supermodular *H* or in the $\left(H_{1},H_{\infty }\right)$-capacity ordering. Moreover, this work establishes some relationships of these new structural preorders with other existing preorders from the literature.

As an example application, we used channel-supermodularity to prove an optimality result of a greedy query anonymization algorithm.

It is our belief that the connection between supermodular functions and some commonly used entropy measures, made in Section 3.1, will prove useful for posterior investigations in information theory (for example, given the vast literature of supermodular functions over Euclidean space [50] (Chapter 6.D) and [49]). Further directions of work include investigating other useful properties of channel-supermodular entropies, and further applications of channel-supermodularity to anonymity.

## Figures and Tables

**Table 1:** Some examples of core-concave entropies.

	η(r)	F(p)		Conditional Form H(X|Y)
Shannon $\left(H_{1}\right)$	*r*	$-\sum_{i}p_{i}\log\left(p_{i}\right)$		$\sum_{y}p\left(y\right)H_{1}\left(X|y\right)$
Min-entropy $\left(H_{\infty }\right)$	−log(−r)	$-\max_{i}p_{i}$		$-\log\left(\sum_{y}p\left(y\right)\max_{x}p_{X|y}\left(x\right)\right)$
Guessing [44] $\left(H_{G}\right)$	*r*	$\sum_{i}ip_{\left[i\right]}$		$\sum_{y}p\left(y\right)H_{G}\left(X|y\right)$
Arimoto–Rényi [43] $\left(H_{\alpha }\right)$	$\frac{\alpha }{1-\alpha }\log\left(r\right)$	${∥p∥}_{\alpha }$	if 0<α<1	$\frac{\alpha }{1-\alpha }\log\left(\sum_{y}p\left(y\right){∥p_{X|y}∥}_{\alpha }\right)$
	$\frac{\alpha }{1-\alpha }\log\left(-r\right)$	$-{∥p∥}_{\alpha }$	if α>1	
Hayashi–Rényi [47] $\left(H_{\alpha }^{\prime }\right)$	$\frac{1}{1-\alpha }\log\left(r\right)$	${∥p∥}_{\alpha }^{\alpha }$	if 0<α<1	$\frac{1}{1-\alpha }\log\left(\sum_{y}p\left(y\right){∥p_{X|y}∥}_{\alpha }^{\alpha }\right)$
	$\frac{1}{1-\alpha }\log\left(-r\right)$	$-{∥p∥}_{\alpha }^{\alpha }$	if α>1	
Tsallis [45] $\left(H_{\left(\alpha ,\alpha \right)}\right)$	$\frac{1}{\alpha -1}\left(1-r\right)$	${∥p∥}_{\alpha }^{\alpha }$	if 0<α<1	$\frac{1}{\alpha -1}\left(1-\left(\sum_{y}p\left(y\right){∥p_{X|y}∥}_{\alpha }^{\alpha }\right)\right)$
	$\frac{1}{\alpha -1}\left(1+r\right)$	$-{∥p∥}_{\alpha }^{\alpha }$	if α>1	
Sharma–Mittal [46] $\left(H_{\left(\alpha ,\beta \right)}\right)$	$\frac{1}{\beta -1}\left(1-r^{\frac{1-\beta }{1-\alpha }}\right)$	${∥p∥}_{\alpha }^{\alpha }$	if 0<α<1	$\frac{1}{\beta -1}\left(1-{\left(\sum_{y}p\left(y\right){∥p_{X|y}∥}_{\alpha }^{\alpha }\right)}^{\frac{1-\beta }{1-\alpha }}\right)$
	$\frac{1}{\beta -1}\left(1-{\left(-r\right)}^{\frac{1-\beta }{1-\alpha }}\right)$	$-{∥p∥}_{\alpha }^{\alpha }$	if α>1	

## Data Availability

Not applicable.

## Appendix A. Proofs of Section 3

### Appendix A.1. Proof of Proposition 2

(1) For Shannon entropy, Definition 5 yields

$$G_{F}\left(r\right)=-{∥r∥}_{1}\sum_{i}^{n}\frac{r_{i}}{{∥r∥}_{1}}\log\left(\frac{r_{i}}{{∥r∥}_{1}}\right)=-\sum_{i}^{n}r_{i}\log\left(\frac{r_{i}}{{∥r∥}_{1}}\right).$$

Suppose $r\in R_{>0}^{n}$. Then, for all i,j with i≠j we have

(A1) $$\frac{\partial^{2}G_{F}\left(r\right)}{\partial r_{i}\partial r_{j}}\;=\;\frac{1}{{∥r∥}_{1}\ln\left(2\right)}\geq 0.$$

Supermodularity of $G_{F}$ restricted to $r\in R_{>0}^{n}$ then follows from (4). If, however, $r\in R_{\geq 0}^{n}\backslash R_{>0}^{n}$, the second derivative may not exist. In such a case, supermodularity can be established by a limiting argument. Let ϵ>0 and $r^{\prime }=r+\epsilon \left(\sum_{k}e_{k}\right)$.

Then (4) and (A1) imply that

$$G_{F}\left(r^{\prime }+\delta_{1}e_{i}+\delta_{2}e_{j}\right)+G_{F}\left(r^{\prime }\right)\geq G_{F}\left(r^{\prime }+\delta_{1}e_{i}\right)+G_{F}\left(r^{\prime }+\delta_{2}e_{j}\right).$$

As $G_{F}$ is continuous, taking ϵ→0 in both sides of the inequality above, we obtain

$$G_{F}\left(r+\delta_{1}e_{i}+\delta_{2}e_{j}\right)+G_{F}\left(r\right)\geq G_{F}\left(r+\delta_{1}e_{i}\right)+G_{F}\left(r+\delta_{2}e_{j}\right).$$

Thus, $G_{F}$ is supermodular following (3).

(2) For Arimoto–Rényi entropies of order α∈(0,1), we have

$$G_{F}\left(r\right)={∥r∥}_{1}F\left(\frac{r}{{∥r∥}_{1}}\right)={∥r∥}_{1}{∥\frac{r}{{∥r∥}_{1}}∥}_{\alpha }={∥r∥}_{\alpha }$$

and similarly, $G_{F}\left(r\right)=-{∥r∥}_{\alpha }$ for entropies of order α>1.

Now, for all $r\in R_{>0}^{n}$ and i,j with i≠j, we have

$$\frac{\partial^{2}{∥r∥}_{\alpha }}{\partial r_{i}\partial r_{j}}\;=\;\left(1-\alpha \right)r_{i}^{\alpha -1}r_{j}^{\alpha -1}{\left(\sum_{k=1}^{n}r_{k}^{\alpha }\right)}^{\frac{1}{\alpha }-2}$$

which is negative if α>1 and positive if 0<α<1. Thus, from Equation (4), $G_{F}$ restricted to $R_{>0}^{n}$ is supermodular for all α∈(0,1)∪(1,∞). Supermodularity of $G_{F}$ can then be established by a limiting argument similar to the one in the proof for Shannon entropy.

(3) For the *k*-tries entropy, we have

$$G_{F}\left(r\right)=-{∥r∥}_{1}\left(\sum_{i=1}^{k}\frac{r_{\left[i\right]}}{{∥r∥}_{1}}\right)=-\sum_{i=1}^{k}r_{\left[i\right]}.$$

Let $r\in R_{\geq 0}^{n}$, let i,j be such that i≠j and let $\delta_{1},\delta_{2}>0$. From Equation (3), it suffices to prove that

$$G_{F}\left(r+\delta_{1}e_{i}\right)-G_{F}\left(r\right)\leq G_{F}\left(r+\delta_{1}e_{i}+\delta_{2}e_{j}\right)-G_{F}\left(r+\delta_{2}e_{j}\right).$$

Let $r^{\prime }=r+\delta_{1}e_{i}$, $r^{\prime \prime }=r+\delta_{2}e_{j}$ and $r^{\prime \prime \prime }=r+\delta_{1}e_{i}+\delta_{2}e_{j}$. The inequality above is equivalent to

(A2) $$\sum_{l=1}^{k}r_{\left[l\right]}^{\prime }-\sum_{l=1}^{k}r_{\left[l\right]}\geq \sum_{l=1}^{k}r_{\left[l\right]}^{\prime \prime \prime }-\sum_{l=1}^{k}r_{\left[l\right]}^{\prime \prime }.$$

If $r_{i}+\delta_{1}$ is not amongst the *k* largest elements in $r^{\prime \prime \prime }$, then the right-hand side of (A2) is equal to 0, and there is nothing to prove. If $r_{i}+\delta_{1}$ is amongst the *k* largest elements in $r^{\prime \prime \prime }$, then it is also amongst the *k* largest elements in $r^{\prime }$, and either:

- $r_{i}$ is amongst the *k* largest elements in r and $r^{\prime \prime }$, and thus both sides of (A2) are equal to $\delta_{1}$;
- $r_{i}$ is amongst the *k* largest elements in r but not in $r^{\prime \prime }$. In which case the left-hand side of (A2) is equal to $\delta_{1}$, and the right-hand side of (A2) is less than $\delta_{1}$;
- $r_{i}$ not amongst the *k* largest elements in r and neither in $r^{\prime \prime }$. In this case the left-hand side of (A2) is larger then the right-hand side, as the *k*th largest element of $r^{\prime \prime }$ is greater or equal than the *k*th largest element of r.

In any case, (A2) holds.

(4) For guessing entropy, Definition 5 yields

$$G_{F}\left(r\right)={∥r∥}_{1}\sum_{i}\frac{ir_{\left[i\right]}}{{∥r∥}_{1}}=\sum_{i}ir_{\left[i\right]}.$$

Let i,j be such that i≠j and let $\delta_{1},\delta_{2}>0$. Suppose, without loss of generality, that $r_{i}+\delta_{1}\geq r_{j}+\delta_{2}$. We will prove that

(A3) $$G_{F}\left(r+\delta_{1}e_{i}+\delta_{2}e_{j}\right)-G_{F}\left(r+\delta_{1}e_{i}\right)\geq G_{F}\left(r+\delta_{2}e_{j}\right)-G_{F}\left(r\right).$$

Let $I_{r+\delta_{1}e_{i}}=\left\{k\leq n\;|\;r_{j}<r_{k}<r_{j}+\delta_{2}\right\}$—that is, $I_{r+\delta_{1}e_{i}}$ is the set of coordinates of $r+\delta_{1}e_{i}$ whose value is between $r_{j}$ and $r_{j}+\delta_{2}$.

Let m−1 be the number of entries of $r+\delta_{1}e_{i}+\delta_{1}e_{j}$ that are greater than or equal to $r_{j}+\delta_{2}$. Then, there are $\left(m+|I_{r+\delta_{1}e_{i}}|-1\right)$ coordinates of $r+\delta_{1}e_{i}$ that are strictly greater than $r_{j}$.

The difference $G_{F}\left(r+\delta_{1}e_{i}+\delta_{2}e_{j}\right)-G_{F}\left(r+\delta_{1}e_{i}\right)$ is then $m\left(r_{j}+\delta_{2}\right)-(m+|I_{p+\delta_{1}e_{i}}\left|\right)r_{j}$, plus the sum of the entries given by the set $I_{p+\delta_{1}e_{i}}$, as the integers by which they are multiplied are decreased by one. As we assume $r_{i}+\delta_{1}\geq r_{j}+\delta_{2}$, $i\notin I_{p+\delta_{1}e_{i}}$, and the value of the right hand-side of (A3) reduces to

$$\begin{array}{cc} \; & G_{F}\left(r+\delta_{1}e_{i}+\delta_{2}e_{j}\right)-G_{F}\left(r+\delta_{1}e_{i}\right)\\ = & m\left(r_{j}+\delta_{2}\right)-(m+|I_{r+\delta_{1}e_{i}}\left|\right)r_{j}+\sum_{k\in I_{r+\delta_{1}e_{i}}}r_{k}\\ = & -|I_{r+\delta_{1}e_{i}}|r_{j}+m\delta_{2}+\sum_{k\in I_{r+\delta_{1}e_{i}}}r_{k}. \end{array}$$

We now turn to the right-hand side of (A3), the difference $G_{F}\left(p+\delta_{2}e_{j}\right)-G_{F}\left(p\right)$. We define $I_{p}$ similarly to $I_{p+\delta_{1}e_{i}}$ and divide the proof in three cases.

*Case 1* ($r_{i}\geq r_{j}+\delta_{2}$): In this case, there are m−1 entries of $r+\delta_{2}e_{j}$ greater or equal to $r_{j}+\delta_{2}$, and $\left(m+|I_{r}|-1\right)$ entries of r strictly greater than $r_{j}$. Moreover, $I_{r}=I_{r+\delta_{1}e_{i}}$, and we obtain

$$\begin{array}{cc} \; & G_{F}\left(r+\delta_{2}e_{j}\right)-G_{F}\left(r\right)\\ = & m\left(r_{j}+\delta_{2}\right)-(m+|I_{r}\left|\right)r_{j}+\sum_{k\in I_{r}}r_{k}\\ = & G_{F}\left(r+\delta_{1}e_{i}+\delta_{2}e_{j}\right)-G_{F}\left(r+\delta_{1}e_{i}\right). \end{array}$$

*Case 2* ($r_{j}<r_{i}<r_{j}+\delta_{2}$): In this case, there are m−1 entries of $r+\delta_{2}e_{j}$ greater or equal to $r_{j}+\delta_{2}$, and $\left(m+\;|I_{r}|-2\right)$ entries of r strictly greater than $r_{j}$. This time, $I_{r}=I_{r+\delta_{1}e_{i}}\cup \left\{i\right\}$, and we have

$$\begin{array}{cc} \; & G_{F}\left(r+\delta_{2}e_{j}\right)-G_{F}\left(r\right)\\ = & \left(m-1\right)\left(r_{j}+\delta_{2}\right)+\sum_{k\in I_{r}}r_{k}-(m-1+|I_{r}\left|\right)r_{j}\\ = & \left(m-1\right)\left(r_{j}+\delta_{2}\right)+\sum_{k\in I_{r+\delta_{1}e_{i}}}r_{k}+r_{i}-(m+|I_{r+\delta_{1}e_{i}}\left|\right)r_{j}\\ = & -|I_{r+\delta_{1}e_{i}}|r_{j}+m\delta_{2}+\sum_{k\in I_{r+\delta_{1}e_{i}}}r_{k}+r_{i}-\left(r_{j}+\delta_{2}\right)\\ \leq & -|I_{r+\delta_{1}e_{i}}|r_{j}+m\delta_{2}+\sum_{k\in I_{r+\delta_{1}e_{i}}}r_{k}\\ = & G_{F}\left(r+\delta_{1}e_{i}+\delta_{2}e_{j}\right)-G_{F}\left(r+\delta_{1}e_{i}\right), \end{array}$$

where the inequality comes from the assumption that $r_{i}<r_{j}+\delta_{2}$.

*Case 3* ($r_{i}\leq r_{j}$): In this case, again there are m−1 entries of $r+\delta_{2}e_{j}$ greater or equal to $r_{j}+\delta_{2}$, and $\left(m+\;|I_{r}|-2\right)$ entries of r strictly greater than $r_{j}$. This time, $I_{r}=I_{r+\delta_{1}e_{i}}$, and we have

$$\begin{array}{cc} \; & G_{F}\left(r+\delta_{2}e_{j}\right)-G_{F}\left(r\right)\\ = & \left(m-1\right)\left(r_{j}+\delta_{2}\right)+\sum_{k\in I_{r}}r_{k}-(m-1+|I_{r}\left|\right)r_{j}\\ = & -|I_{r}|r_{j}+\left(m-1\right)\delta_{2}+\sum_{k\in I_{r}}r_{k}\\ \leq & -|I_{r}|r_{j}+m\delta_{2}+\sum_{k\in I_{r}}r_{k}\\ = & G_{F}\left(r+\delta_{1}e_{i}+\delta_{2}e_{j}\right)-G_{F}\left(r+\delta_{1}e_{i}\right). \end{array}$$

Therefore, $G_{F}$ is supermodular.

### Appendix A.2. Proof of Proposition 3

(1) For all these entropies, Definition 5 yields

$$G_{F}\left(r\right)={∥r∥}_{1}{∥\frac{r}{{∥r∥}_{1}}∥}_{\alpha }^{\alpha }={∥r∥}_{\alpha }^{\alpha }{∥r∥}_{1}^{\left(1-\alpha \right)},$$

if 0<α<1, and $G_{F}\left(r\right)=-{∥r∥}_{\alpha }^{\alpha }{∥r∥}_{1}^{\left(1-\alpha \right)}$ if α>1.

Now, if $r\in R_{>0}^{n}$, we have

$$\frac{\partial^{2}{∥r∥}_{\alpha }^{\alpha }{∥r∥}_{1}^{\left(1-\alpha \right)}}{\partial r_{i}\partial r_{j}}\;=\;\alpha \left(1-\alpha \right){∥r∥}_{1}^{-\alpha }\left(r_{i}^{\alpha -1}+r_{j}^{\alpha -1}-{∥r∥}_{\alpha }^{\alpha }{∥r∥}_{1}^{-1}\right),$$

and by (4), $G_{F}$ is supermodular only if the partial derivative above is positive if α∈(0,1) and negative if α>1. Equivalently, if $G_{F}$ is supermodular then for all $r\in R_{>0}^{n}$,

(A4) $$\forall i,j\;\mathrm{such}\;\mathrm{that}\;i\neq j\;r_{i}^{\alpha -1}+r_{j}^{\alpha -1}-{∥r∥}_{\alpha }^{\alpha }{∥r∥}_{1}^{-1}\geq 0.$$

Thus, it suffices to provide, for each α, one vector r such that (A4) does not hold.

*Case 1*(α∈(0,1)): Let $r\in R_{>0}^{n}$ be the vector with coordinates $r_{1}=r_{2}=1$ and $r_{j}=\epsilon$ for all j>2. Then,

$$r_{1}^{\alpha -1}+r_{2}^{\alpha -1}-{∥r∥}_{\alpha }^{\alpha }{∥r∥}_{1}^{-1}=2-\frac{2+\left(n-2\right)\epsilon^{\alpha }}{2+(n-2)\epsilon }.$$

Now, $\lim_{n\to \infty }\frac{2+\left(n-2\right)\epsilon^{\alpha }}{2+(n-2)\epsilon }=\epsilon^{\alpha -1}$, and $\epsilon^{\alpha -1}$ can be made arbitrarily large by choosing a suitably small ϵ. Thus, for an appropriate choice of *n* and ϵ, $\frac{2+\left(n-2\right)\epsilon^{\alpha }}{2+(n-2)\epsilon }>2$, and (A4) does not hold.

*Case 2*(α>1): Fix n>2. Let α>1, ϵ>0 and pick a>0 such that

$$a^{\alpha }-2a-2>\left(n-3\right)\left(2\epsilon -\epsilon^{\alpha }\right).$$

Notice that such *a* is guaranteed to exist because α>1. Let $r\in R_{>0}^{n}$ be the vector with $r_{1}=r_{2}=1$, $r_{3}=a$ and $r_{k}=\epsilon$ for all k>3. Then

$$r_{1}^{\alpha -1}+r_{2}^{\alpha -1}-{∥r∥}_{\alpha }^{\alpha }{∥r∥}_{1}^{-1}=2-\frac{2+a^{\alpha }+\left(n-3\right)\epsilon^{\alpha }}{2+a+(n-3)\epsilon }=\frac{2+2a-a^{\alpha }+\left(n-3\right)\left(2\epsilon -\epsilon^{\alpha }\right)}{2+a+(n-3)\epsilon }<0.$$

and (A4) does not hold. Therefore, $G_{F}$ is not supermodular.

(2) By Definition 5, we have

$$G_{F}\left(r\right)=-\max_{A\in P}\sum_{i\in A}r_{i}.$$

Let n=3, P={{1,2},{3}}, and let r=(3,1,3), $s=\left(1,3,3\right)\in R_{\geq 0}^{3}$. Then,

$$G_{F}\left(r\vee s\right)+G_{F}\left(r\wedge s\right)=-9<-8=G_{F}\left(r\right)+G_{F}\left(s\right).$$

## Appendix B. Proofs of Section 5

### Appendix B.1. Proof of Proposition 5

(1) follows immediately from Definition 9, and (2) from Proposition 4.2. Statement (3) is proven by the following channels:

$$\left(\begin{array}{c} 1\\ 1 \end{array}\right)\geq_{d}\left(\begin{array}{cc} 0.5 & 0.5\\ 0.5 & 0.5 \end{array}\right).$$

For (4), consider the channels $K_{1}$, $K_{2}$ as follows:

100112121012121212.

These channels were introduced in [3], where it is proved that $K_{1}{≱}_{\ln}^{H_{1}}K_{2}$. However $K_{1}\geq_{\mathrm{ds}}K_{2}$, as

10011212≥d10001212121414≥s10012012121414≥d1012121212.

Statement (5) follows from (1) and from Theorem 2.

To prove (6), consider the following channels, for some r∈(0,1) and δ,ϵ>0:

K1=r1−rr+δ1−r−δK2=K11+ϵ−ϵ1212.

Notice that $K_{1}$ and $K_{2}$ are indeed channels if δ and ϵ are small enough. These channels were introduced in [3] as an example for which (for δ,ϵ small enough) $K_{1}\geq_{\ln}^{H_{1}}K_{2}$ but $K_{1}{≱}_{d}K_{2}$. As their input set is of size 2, Proposition 4.1 implies that $K_{1}{≱}_{\mathrm{ds}}K_{2}$.

Statement (7) follows directly from Definition 9, Proposition 1, and Theorem 4.

For (8), consider the following $K_{1}$, $K_{2}$:

01252425120121313131250242512012131313.

In this case, $K_{2}={⋄}_{1,2}K_{1}$, and thus $K_{1}\geq_{\mathrm{ds}}K_{2}$. Suppose, to derive a contradiction, that $K_{1}\geq_{\mathrm{sh}}K_{2}$ Then there is a finite collection $\left\{\left(g_{i},T_{i},R_{i}\right)\right\}$ such that each $T_{i}$, $R_{i}$ is a deterministic channel, each $g_{i}>0$, $\sum g_{i}=1$ and $K_{2}=\sum_{i}g_{i}T_{i}K_{1}R_{i}$. Notice that, for all *i*, we must have

(A5) $$\left(T_{i}K_{1}R_{i}\right)\left(y_{2}|x_{1}\right)=\left(T_{i}K_{1}R_{i}\right)\left(y_{2}|x_{2}\right)=0.$$

Let I={i|(TiK1Ri)(y1|x1)≤125}. We claim that

(A6) $$\mathrm{for}\;\mathrm{all}\;i\in I,\;\left(T_{i}K_{1}R_{i}\right)\left(y_{1}|x_{3}\right)+\left(T_{i}K_{1}R_{i}\right)\left(y_{3}|x_{2}\right)+\left(T_{i}K_{1}R_{i}\right)\left(y_{3}|x_{3}\right)\geq \frac{4}{3}.$$

To see why, notice that channels of the form $T_{i}K_{1}$ are those channels whose rows are equal to rows of $K_{1}$ (with possible reordering and duplicates), and channels of the form $\left(T_{i}K_{1}\right)R_{i}$ are those obtaining by permutating or summing columns of $\left(T_{i}K_{1}\right)$.

If the first row of $T_{i}K_{1}$ is either (12,0,12) or (13,13,13). Then, in order that i∈I, the third column of $T_{i}K_{1}R_{i}$ must be either (1) equal to the sum of the first and third columns of $T_{i}K_{1}$, (2) or equal to the sum of all columns of $T_{i}K_{1}$. In either case, we have (TiK1Ri)(y3|x2)≥23 and (TiK1Ri)(y3|x3)≥23, so (A6) holds.

If the first row of $T_{i}K_{1}$ is (0,125,2425), the proof is not as straightforward, and we divide it into two subcases. The first subcase is when the second row is either (13,13,13) or (12,0,12), then each column of $T_{i}K_{1}$ will be mapped to the first or to the third column of $T_{i}K_{1}R_{i}$, and therefore $\left(T_{i}K_{1}R_{i}\right)\left(y_{1}|x_{3}\right)+\left(T_{i}K_{1}R_{i}\right)\left(y_{3}|x_{3}\right)=1$. Then, (A6) follows from observing that (TiK1Ri)(y3|x2)≥13. The second subcase to consider is when the second row is (0,125,2425). In this case, we have (TiK1Ri)(y3|x2)≥2425, and, by considering each of the three possible choices for the third row of $T_{i}K_{1}$, it is easy to see that (TiK1Ri)(y1|x3)+(TiK1Ri)(y3|x3)≥12.

As K2(y1|x3)+K2(y3|x2)+K2(y3|x3)=76, it follows from (A6) that

$$\begin{array}{cc} \frac{7}{6} & =\sum_{i}g_{i}\left(\left(T_{i}K_{1}R_{i}\right)\left(y_{1}|x_{3}\right)+\left(T_{i}K_{1}R_{i}\right)\left(y_{3}|x_{2}\right)+\left(T_{i}K_{1}R_{i}\right)\left(y_{3}|x_{3}\right)\right)\\ \; & \geq \sum_{i\in I}g_{i}\left(\left(T_{i}K_{1}R_{i}\right)\left(y_{1}|x_{3}\right)+\left(T_{i}K_{1}R_{i}\right)\left(y_{3}|x_{2}\right)+\left(T_{i}K_{1}R_{i}\right)\left(y_{3}|x_{3}\right)\right)\\ \; & \geq \frac{4}{3}\sum_{i\in I}g_{i}. \end{array}$$

Thus, it follows that ∑i∈Igi≤78, and therefore ∑i∉Igi≥18. Since

mini∉I(TiK1Ri)(y1|x1)≥13, we have

$$\begin{array}{cc} \frac{1}{25}=K_{2}\left(y_{1}|x_{1}\right) & =\sum_{i=1}^{k}g_{i}\left(T_{i}K_{1}R_{i}\right)\left(y_{1}|x_{1}\right)\\ \; & \geq \sum_{i\notin I}g_{i}\left(T_{i}K_{1}R_{i}\right)\left(y_{1}|x_{1}\right)\\ \; & \geq \frac{1}{3}\sum_{i\notin I}g_{i}\\ \; & \geq \frac{1}{3}\times \frac{1}{8}=\frac{1}{24} \end{array}$$

which is absurd.

For (9), let $K_{1}$, $K_{2}$ be the following channels:

$$\left(\begin{array}{cc} 1 & 0\\ 1 & 0\\ 0 & 1 \end{array}\right)\;\left(\begin{array}{cc} 1 & 0\\ 0 & 1\\ 1 & 0 \end{array}\right).$$

Notice that $K_{2}$ is obtained from $K_{1}$ by row permutation; hence, $K_{1}\geq_{\mathrm{sh}}K_{2}$. However, $K_{1}{≱}_{\mathrm{mc}}^{H_{1}}K_{2}$ (take, for example, pX=(12,12,0)). Thus, (7) yields $K_{1}{≱}_{\mathrm{ds}}K_{2}$.

### Appendix B.2. Proof of Proposition 9

(1) Follows from Lemma 1 and Shannon’s result in [1].

(2) In light of Lemma 1, we need only to prove that $K_{1}\geq_{\mathrm{sh}}K_{2}\Rightarrow K_{1}\geq_{c}^{H_{\infty }}K_{2}$.

Firstly, we prove that, given channels $W_{1}$, $W_{2}$ and $W_{3}=tW_{1}+\left(1-t\right)W_{2}$, we have $C_{H_{\infty }}\left(W_{3}\right)$$\leq \max(C_{H_{\infty }}\left(W_{1}\right),C_{H_{\infty }}\left(W_{2}\right))$. Keeping in mind the identity $C_{H_{\infty }}\left(W_{3}\right)=\log$$\sum_{y}\max_{x}W_{3}\left(x,y\right)$ ([43] Remark 3),

$$\begin{array}{cc} C_{H_{\infty }}\left(W_{3}\right)= & \log\left(\sum_{y}\max_{x}\left(tW_{1}\left(x,y\right)+\left(1-t\right)W_{2}\left(x,y\right)\right)\right)\\ \leq & \log\left(t\sum_{y}\max_{x}W_{1}\left(x,y\right)+\left(1-t\right)\sum_{y}\max_{x}W_{2}\left(x,y\right)\right)\\ \leq & \max(C_{H_{\infty }}\left(W_{1}\right),C_{H_{\infty }}\left(W_{2}\right)) \end{array}$$

where the last inequality follows from quasi-convexity of log. Now, if $K_{2}=\left(\sum_{i}g_{i}R_{i}K_{1}T_{i}\right)$, then $C_{H_{\infty }}\left(K_{2}\right)=C_{H_{\infty }}\left(\sum_{i}g_{i}R_{i}K_{1}T_{i}\right)\leq \max_{i}C_{H_{\infty }}\left(R_{i}K_{1}T_{i}\right)\leq C_{H_{\infty }}\left(K_{1}\right)$, where the last inequality follows from ([33] Theorem 6).

(3) This is readily seen from the fact that $\geq_{c}^{H_{1}}$ and $\geq_{c}^{H_{\infty }}$ are total preorders that do not coincide. To make the argument concrete, consider channels $K_{1}$, $K_{2}$ as follows:

121200121212012252515152525251525.

Then, from ([53] Theorem 2.7.1) $C_{H_{\infty }}\left(K_{1}\right)>C_{H_{1}}\left(K_{2}\right)$, whereas, from ([43] Remark 3), $C_{H_{\infty }}\left(K_{1}\right)<C_{H_{\infty }}\left(K_{2}\right)$. Thus, (1) and (2) imply that $K_{1}{≱}_{\mathrm{shs}}K_{2}$ and $K_{2}{≱}_{\mathrm{shs}}K_{1}$.
